# Analysis of Copper Welding Parameters during the Manufacture of Tubular Profiles Using Unconventional Extrusion Processes

**DOI:** 10.3390/ma17194737

**Published:** 2024-09-27

**Authors:** Marcin Knapiński, Teresa Bajor, Anna Kawałek, Grzegorz Banaszek

**Affiliations:** Faculty of Production Engineering and Materials Technology, Czestochowa University of Technology, 42-201 Czestochowa, Poland; marcin.knapinski@pcz.pl (M.K.); anna.kawalek@pcz.pl (A.K.); grzegorz.banaszek@pcz.pl (G.B.)

**Keywords:** FEM, copper, tube, extrusion, bridge die, Conform

## Abstract

In recent years, there has been a lack of information in the literature regarding the extrusion and connection of closed profiles from oxygen-free copper in bridge dies. Available studies contain information on the processes of extrusion and connection of profiles from aluminium alloys and various types of steel. However, there is a lack of detailed data on the values of technological parameters for which copper is joined in the extrusion process. Therefore, one of the goals of this work is to fill the gap in the literature regarding the extrusion of oxygen-free copper in bridge dies. In this work, the authors determined the thermo-mechanical conditions at which oxygen-free copper will be joined. This paper describes the effects of charge temperature and hydrostatic pressure in the weld zone of a bridge die on copper bonding in the fabrication of tubular profiles. Physical tests of the welding process under the conditions of upsetting a material consisting of two parts were carried out using the Gleeble 3800 metallurgical process simulator with the PocketJaw module in the standard configuration for SICO (strain-induced crack opening) tests. For the numerical simulations, the commercial computer programme FORGE^®^NxT 2.1. using the finite element method (FEM) was used. Based on the analysis of the test results obtained, it was found that complete material bonding during the extrusion process could be achieved for a charge temperature higher than 600 °C and a hydrostatic pressure of 45–65 MPa.

## 1. Introduction

The hot extrusion process is a popular forming process used in the production of non-ferrous metal products such as wires, tubes, rods, sections, open profiles, closed profiles, etc. [1,2]. The quality and economic demands placed on non-ferrous metal products are forcing companies to look for low-carbon and environmentally friendly solutions, which include extrusion processes [3]. All of these aspects pose a challenge to the manufacturing industry that needs to be addressed in order to develop products with suitable mechanical and physical properties [4]. Extrusion is one of the processes that, in addition to the above advantages, also offers the possibility of greater material utilisation [5]. It is a process that provides high productivity and low energy consumption, and, during its course, the material is subjected to high compressive stresses and strains, meaning that, despite being subjected to a very high degree of plastic processing, it does not lose its integrity [6]. When properly designed and carried out, the extrusion process allows us to obtain products with good mechanical properties [1,2,3,4,5,6].

The extrusion process is widely discussed in the literature with regard to the manufacture of profiles, including large-scale profiles, from aluminium alloys [5]. The method of manufacturing such products makes economic sense in terms of their widespread use. The extrusion process makes it possible to produce a wide variety of structural components in the form of tubes or shaped hollow sections by using bridge dies. The use of a bridge die allows the technological parameters of the process, such as initial charge temperature and press force, to be reduced [7,8,9,10,11]. During extrusion, the charge is divided into several streams of metal and then recombined in the next stage of the process under conditions of high temperature and hydrostatic pressure. The final stage of the process involves obtaining the required finished product [12,13]. The geometrical shape and dimensions of the die bridge and the temperature of the material have a key influence on the method (balance) of metal flow and the strength of the joint. 

A previous study [1] presented the results of a study on the hot extrusion manufacture of round copper rods. This research included microstructure analysis before and after deformation and strength tests. The results showed that the copper rods obtained after the hot extrusion process displayed a homogeneous microstructure and very good mechanical properties.

As with aluminium alloys, tubular (closed) profiles can also be obtained from copper by extrusion. Such processes are used industrially [14]; however, there is a clear lack of information in the literature on the process parameters used during the extrusion of such profiles. In addition, copper alloy hollow profiles are produced in classical extrusion presses, where the charge is preheated to a sufficiently high temperature to carry out the process and then placed in the press chamber and extruded through a die. A significant technological problem is maintaining the temperature parameters of the deformed material under extrusion conditions in unconventional equipment, which includes, for example, the continuous rotary extrusion process, also known as Conform. 

The continuous rotary extrusion process was developed by the late Derek Green of the UK Atomic Energy Authority in 1971 [15]. The equipment he designed had a single rotating wheel with a groove around its circumference. During this process, material is pressed into the groove by a pressure roller and transported downwards via friction between the rotating wheel and the material. The drive wheel is fitted with a movable, groove-closing element with a shaped hole, called a shoe because of its shape. This element has an additional protrusion (called a heel), which closes the groove and serves to collect the material transported by the wheel. Once the “shoe” part of the wheel is closed, the “heel” in it blocks the groove, forcing the material through the hole into the pre-chamber, which is the material buffer, and then into the die which forms the final shape of the product. Continuous rotary extrusion (Conform process) of light metal bars has been a well-known commercial process since the 1970s [15,16,17]. By the turn of the century, this process had become a cost-effective method for extruding copper products. This versatile method enables the manufacture of long products with different cross-sections and virtually unlimited lengths, using round bars as the charge while allowing the microstructure of cast or coarse-grained products to be changed into fine-grained metal (10–20 µm) in a single run. The deformation of copper under such conditions, unlike aluminium, leads to much higher tool pressures and a higher process temperature. It is important to note that the charge material for the process is not preheated and that the increase in temperature is caused by friction against a stationary element called the “shoe”, enclosing the wheel including the material transport groove, and by the high hydrostatic pressure occurring in the material during extrusion. The Conform process has been analysed from a theoretical point of view by Green [15] and Etherington [17]. In turn, Peng et al. [18] carried out the first physical modelling studies of the process by analysing how the material flows in the process using lead. A 2D finite element model for Conform copper extrusion was published in 1993 by Reinikainen et al. [19]. The development of numerical simulation techniques made it possible for a full 3D finite element model to be presented by Velay [20] and Cho [21]. Based on the research results presented in the literature [15,16,17,18,19,20,21,22], it can be concluded that an important factor influencing the potential to produce shaped products using the Conform method is the temperature and the shape of the intermediate chamber, which in the process acts as a material buffer necessary to achieve a uniform infill of the product shaping die. The Conform process is used industrially in the vast majority of cases for the production of solid sections (without internal holes). However, this does not exclude the possibility of using a complex bridge die forming profiles with an internal hole in place of a single-hole die forming the finished product. 

## 2. Aim and Scope of the Work

Given that the temperature of the material entering the final die is determined by the frictional conditions and hydrostatic pressure occurring in the unconventional extrusion process, the authors of this paper attempted to determine the thermal conditions under which pure anaerobic copper should be worked in order to achieve a good quality of bonding of the material separated by the die bridge in the welding chamber of the bridge die. 

Conducting research on the design of the copper extrusion process in a bridge die under industrial conditions is inefficient from both a material- and a time-cost point of view. To this end, the authors proposed using a combination of numerical and physical modelling to determine the technological parameters of the hot extrusion process and the type and shape of the die to ensure the quality of the finished product. 

## 3. Materials and Methodology Used in This Study

The material chosen for the tests in this study was pure anaerobic copper (min. 99.995% Cu; O_2_ < 3 ppm). The mechanical properties of the test material in a cold state are as follows: tensile strength R_m_: 180 N/mm^2^ ± 10 N/mm^2^; elongation A_(200 mm)_: 38% ± 2%.

### 3.1. Methodology for Physical Simulations of Copper Welding 

**Figure 1:**
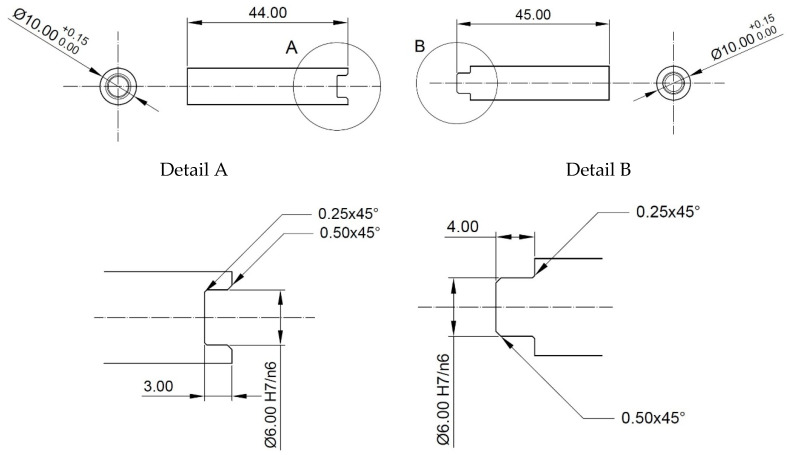
Shapes and dimensions of the specimen used to physically simulate the welding process during plastic deformation.

One of the most significant problems encountered in the manufacture of tubular profiles via bridge die extrusion is knowing the material temperature and hydrostatic pressure values occurring in the weld zone that are sufficient to obtain a permanent joint between two pieces of plastically deformed material. The material used for this study was pure anaerobic copper. The authors of this study carried out physical modelling of the weld process for the test material when upsetting a material consisting of two parts. The modelling used a Gleeble 3800 (Dynamic System Inc., Poestenkill, NY, USA) metallurgical process simulator with the PocketJaw module in the standard configuration for SICO (strain-induced crack opening) tests [23,24,25]. In this configuration, a uniform cylindrical specimen, 86 mm long and 10 mm in diameter, mounted in copper jaws was subjected to the upsetting process. The authors prepared specimens consisting of two parts, the shapes of which are shown in Figure 1.

**Figure 2:**
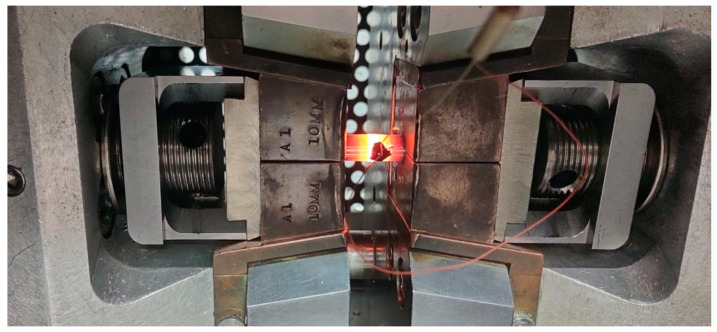
Image of the specimen during the welding experiment at 800 °C.

When developing the test specimen, a tight fit between the ϕ6.00 H7n6 keyway and the mandrel diameter was used (Figure 1 Detail A and Detail B) in order to tightly seal the weld surface before heating the specimen to the strain temperature. Under these conditions, the authors expected to achieve welding of the two parts of the specimens in the central zone of the contact zone with a diameter of approximately 5 mm. To avoid the introduction of oxides or other contaminants into the weld zone immediately prior to the initial joining of the two sample parts, the working surfaces were chemically cleaned with a 60% aqueous nitric acid solution by etching for 3 s. By preparing the specimens in this way for the welding simulation, oxide-free surfaces were obtained in the contact zone of parts A and B, which, under favourable thermomechanical conditions, were welded during the upsetting of the specimen. A K-type thermocouple was mounted onto the surfaces of the above-prepared specimens to measure the surface temperature of the specimen during the experiments. The set was then placed in the testing chamber of the device in jaws suitable for mounting specimens with an external diameter of 10 mm. An image of the specimen placed in the device during the experiment is shown in Figure 2.

**Figure 3:**
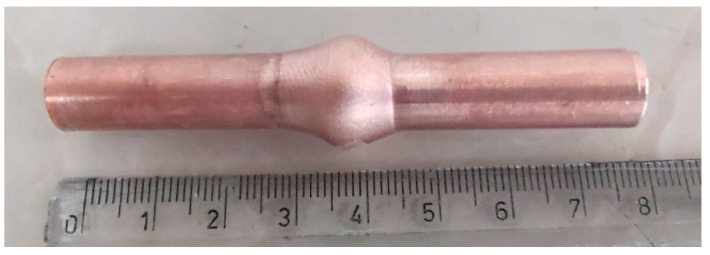
Example of a specimen subjected to upsetting after simulation of the welding process at 660 °C and reduction in length by 10 mm.

The upsetting tests were carried out in a chamber under reduced pressure to limit oxidation of the sample surface. Due to the resistive heating system of the specimen in the jaws, there was a temperature gradient along the length of the specimen, but in the central part of the specimen with a length of about 10 mm, there was a constant temperature throughout the material. Physical simulations were carried out for a total of nine specimens, seven of which were subjected to upsetting which shortened their length by 10 mm at temperatures of 520, 570, 600, 640, 660, 700 and 800 °C, while two were subjected to upsetting which reduced their length by 5 mm at temperatures of 600 and 660 °C. The upsetting time was 0.1 s when the length was reduced by 10 mm and 0.05 s when the length was reduced by 5 mm. Figure 3 shows a image of the specimen after simulation of the welding process at 660 °C and reduction in length by 10 mm.

### 3.2. Numerical Simulation Methodology 

**Figure 4:**
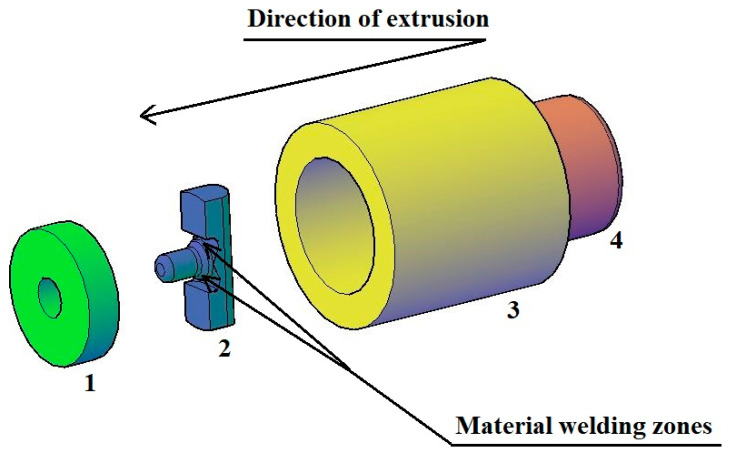
Overview of the extrusion process in an exploded axonometric view with labelling of individual tools.

The geometrical shape and dimensions of the die and die bridge welding chamber were chosen based on a review of the source literature and the authors’ own research in this area. Figure 4 shows an overview of the tools used in the extrusion of a pure anaerobic copper pipe.

The symbol 1 indicates the pipe extrusion die. The diameter of the die bore was 6 mm, while the height of the die was 5 mm. The symbol 2 represents the bridge of the die with the following key dimensions for the extrusion process: the length of the cylindrical part of the mandrel and its diameter were equal to 4 mm. This made it possible to extrude a tube with an external diameter of 6 mm and a wall thickness of 1 mm. The symbol 3 marks the container and the symbol 4 identifies the ram. The welding chambers where the material was bonded after being separated by the die bridge are marked with arrows in Figure 4. 

**Figure 5:**
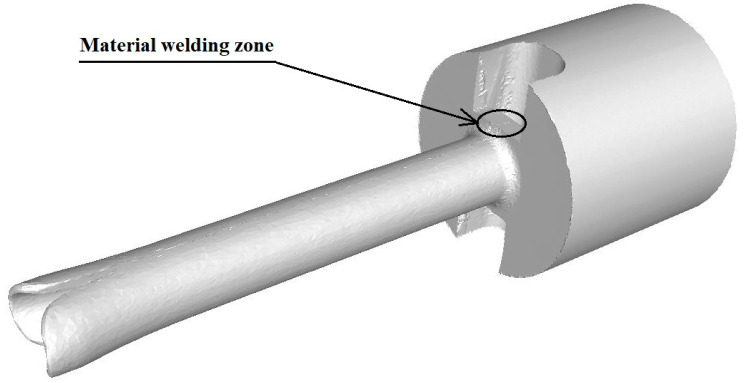
View of the extruded material during the extrusion process.

Figure 5 shows a view of the material during the extrusion process. The arrow marks the zone where material bonding takes place.

The analysis of the extrusion process of the tube was carried out in the commercial computer programme FORGE^®^NxT 2.1. using the finite element method for three cylindrical model specimens 20 mm in diameter and 18 mm in length made from Cu alloy [26]. 

This programme allows the thermomechanical simulation of, among other things, plastic forming processes. A detailed description of the temperature, energy, stress–strain functions, and thermomechanical and friction laws used in the calculations can be found in [27]. 

In this paper, a thermal and viscoplastic deformed model was used to simulate the extrusion process, which is based on large plastic deformation theory. For the generation of the finite element mesh, tetrahedral elements were used. For the numerical simulations, the number of nodes in the volume of the model charge was assumed to be 30,001, while the number of tetrahedral elements was 151,726. 

The value of the friction coefficient between the tool surfaces and the deformed material was equal to μ = 0.3. The heat transfer coefficient between the tools and the material was assumed to be λ = 8100 W/m^2^K, while the heat transfer coefficient between the deformed Cu material and the environment was equal to α = 7 W/m^2^K. 

During the simulation of the extrusion process, the following initial conditions were assumed:

- A ram feed rate of v = 10 mm/s;
- An initial temperature of the model charge in the range of 600–800 °C;
- An ambient temperature of 25 °C and a tool temperature of 350 °C.

## 4. Analysis of the Obtained Research Results

### 4.1. Analysis of the Results of Physical Modelling of the Welding Process

**Figure 6:**
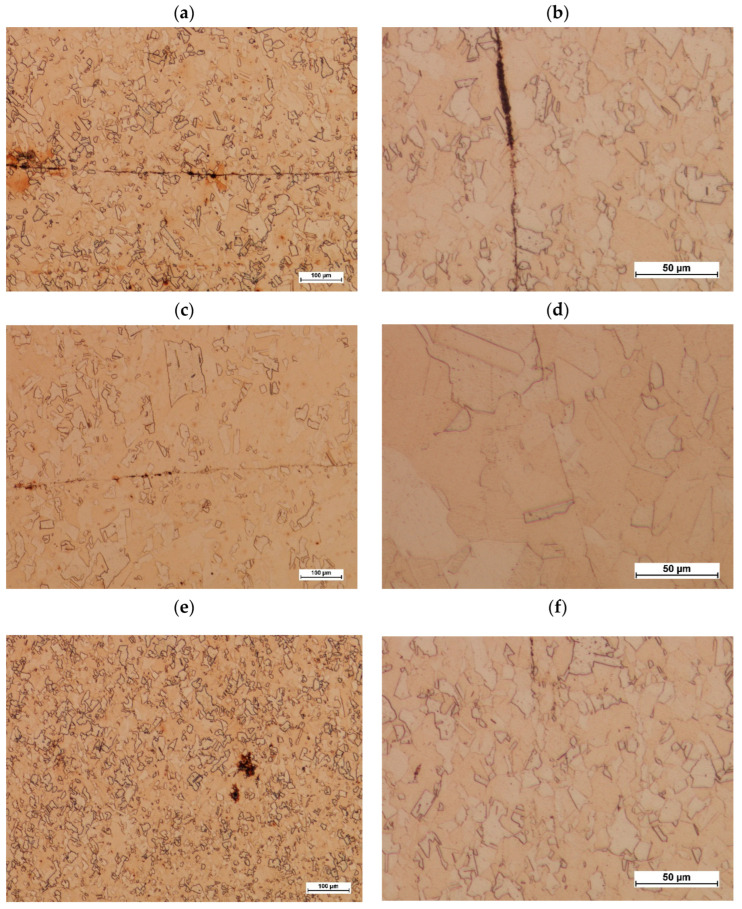
Example images of the bonding zone after the upsetting process: (**a**) at 540 °C and with a 10 mm reduction in length; (**b**) at 540 °C and with a 10 mm reduction in length; (**c**) at 600 °C and with a 5 mm reduction in length; (**d**) at 600 °C and with a 5 mm reduction in length; (**e**) at 640 °C and with a 10 mm reduction in length; (**f**) at 640 °C and with a 10 mm reduction in length.

The deformed specimens were cut along their axes, and metallographic specimens were etched using nital (a mixture of nitric acid and ethyl alcohol) on the cut surfaces. The specimens were observed using a Nikon Eclipse MA200 microscope (Nikon Industrial Metrology, Tokyo, Japan) at various magnifications to identify whether or not a material joint had been achieved. Example images of the bonding zone are shown in Figure 6.

The images of the microstructures shown in Figure 6 confirm that appropriate deformation conditions are necessary to achieve an unbreakable, continuous joint. As can be seen in Figure 6a,b, when deformed at 540 °C and reduced in length by 10 mm, a complete joint was not obtained when observing the front of the specimen. A similar situation can be seen for deformation at 600 °C and a reduction in length of 5 mm (Figure 6c,d). Reducing the length by 10 mm and increasing the temperature to 640 °C resulted in a continuous joint, with no potential discontinuities (Figure 6e,f). Figure 6f shows the microstructure of the fine grains arranged at the probable joint line. The size of the grains varies, but the way they are distributed may be indicative of joint continuity. 

As mentioned earlier, in order to obtain a good-quality welded joint during the plastic deformation of the material, it is necessary to have the right temperature and stress conditions. During the physical simulations, the temperature of the material was continuously controlled, but the stress values occurring in the material interface are a result of its plastic flow resistance and the plastic flow directions of the material. In the upsetting test, there is a state of compression in the direction of the specimen’s axis. The diameter of the specimen increases in the plane of the weld along the radius of the specimen. A good indicator to assess the state of stress occurring in the weld zone is the value of hydrostatic pressure in the volume of the material being deformed. The hydrostatic pressure occurring in a plastically deformed material described by the viscoplastic model is the average stress multiplied by −1 (taken with the inverse sign), calculated as the arithmetic average of the major stresses, and its unit is pascals (Pa). This parameter cannot be measured under the conditions of physical simulation of the process, so additional numerical analyses of the course of the material weld upsetting process were carried out. 

The numerical simulation reproduced the conditions occurring during specimen upsetting in the Gleeble 3800 with the PocketJaw module, in particular the temperature distribution occurring along the specimen axis. Figure 5 shows an example of the simulation results of the upsetting process at a temperature of 600 °C and a reduction in the specimen length by 10 mm.

**Figure 7:**
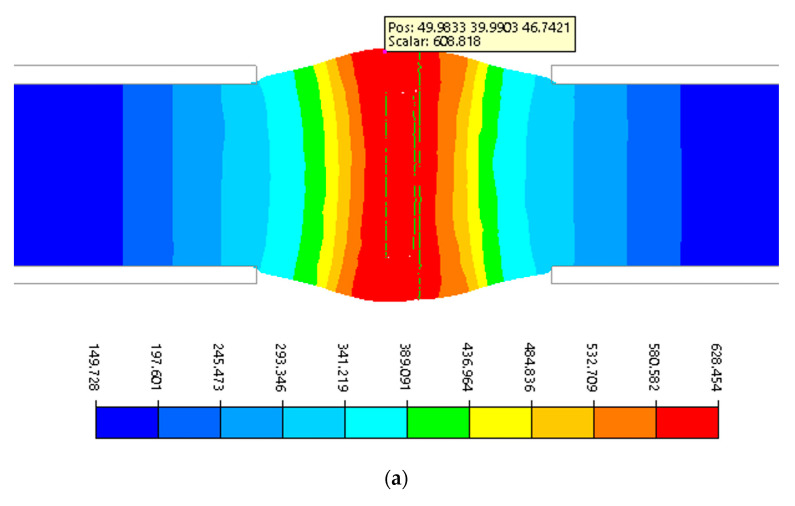

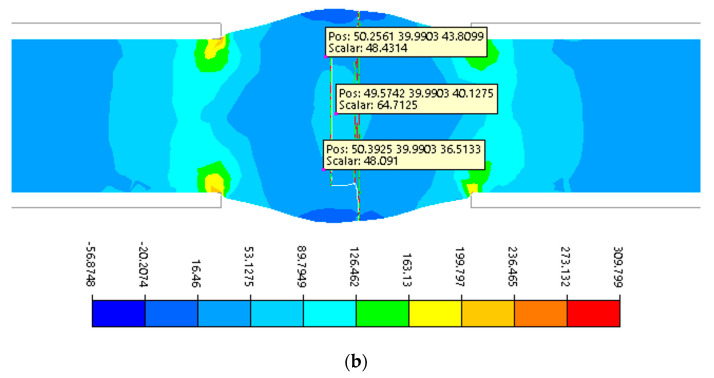
Example results of the numerical simulation of the upsetting process at 600 °C and with a length reduction of 10 mm; (**a**) temperature distribution (°C) in the specimen; (**b**) hydrostatic pressure distribution (MPa) in the weld zone.

The data presented in Figure 7a show that the temperature on the surface of the specimen increased from 600 °C to a value of 608 °C as a result of the deformation process, while in the centre of the specimen, it increased by almost 30 °C. However, it should be noted that, under the conditions of the experiment, the temperature control system maintains its value at a preset level with an accuracy of about 5 °C, so the temperature is overestimated in the numerical simulation results obtained. The data presented in Figure 7b indicate that the hydrostatic pressure reaches a maximum value in the weld zone near the axis of the specimen, and this is about 65 MPa, while it decreases to about 45 MPa with increasing distance from the specimen axis along the radius. With these thermomechanical parameters, according to the analysis of the metallographic specimens, full material bonding was not achieved. Numerical simulations were carried out for four selected temperatures at which partial and full welding of the deformed material was achieved: 570, 600, 640, and 660 °C. A summary of the results of the physical modelling of the welding process of the copper specimens, together with the complementary results of the numerical simulations, is presented in Table 1.

### 4.2. Analysis of Numerical Modelling Research Results

**Figure 8:**
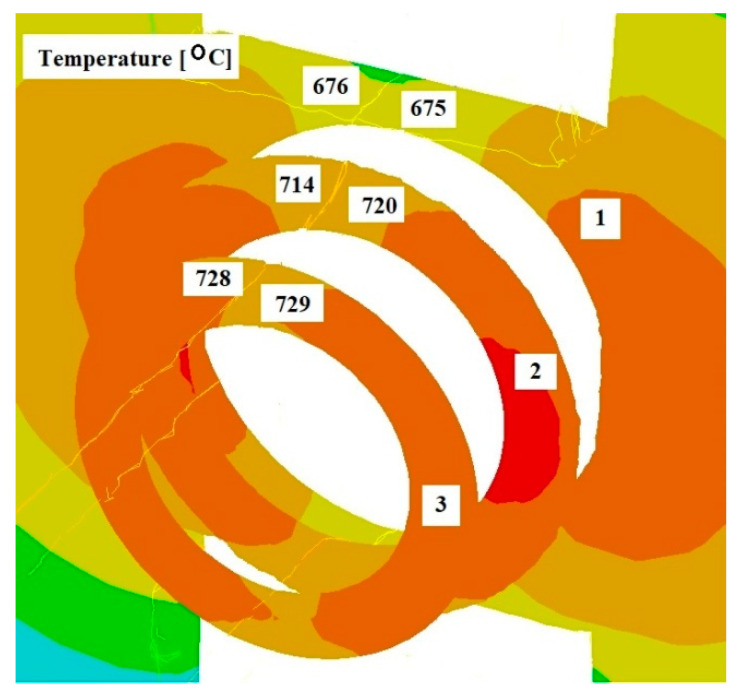
Distribution of temperature values on cross-sections of a charge extruded at 10 mm/s and at 800 °C.

**Figure 9:**
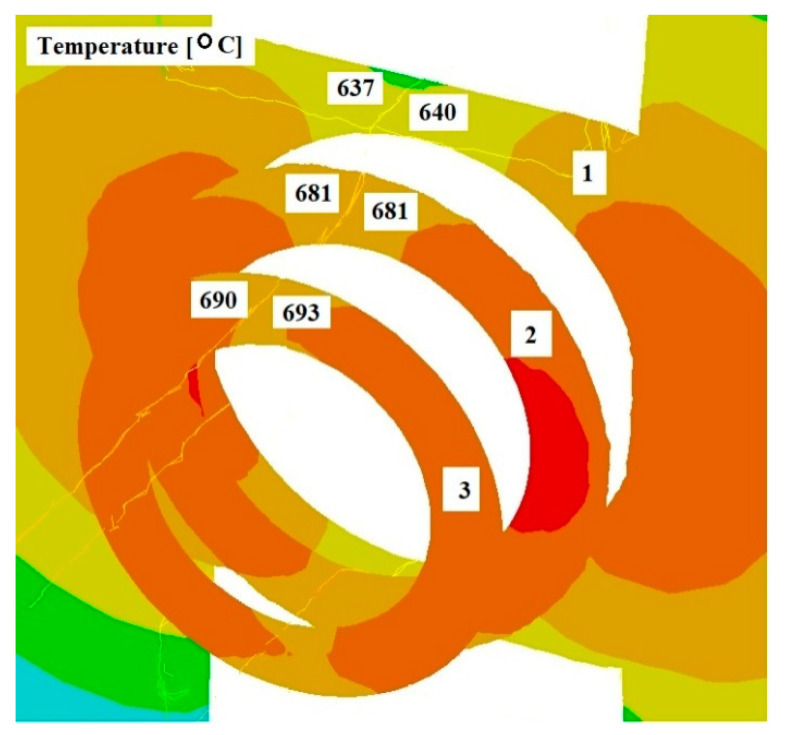
Distribution of temperature values on cross-sections of a charge extruded at 10 mm/s and at 700 °C.

**Figure 10:**
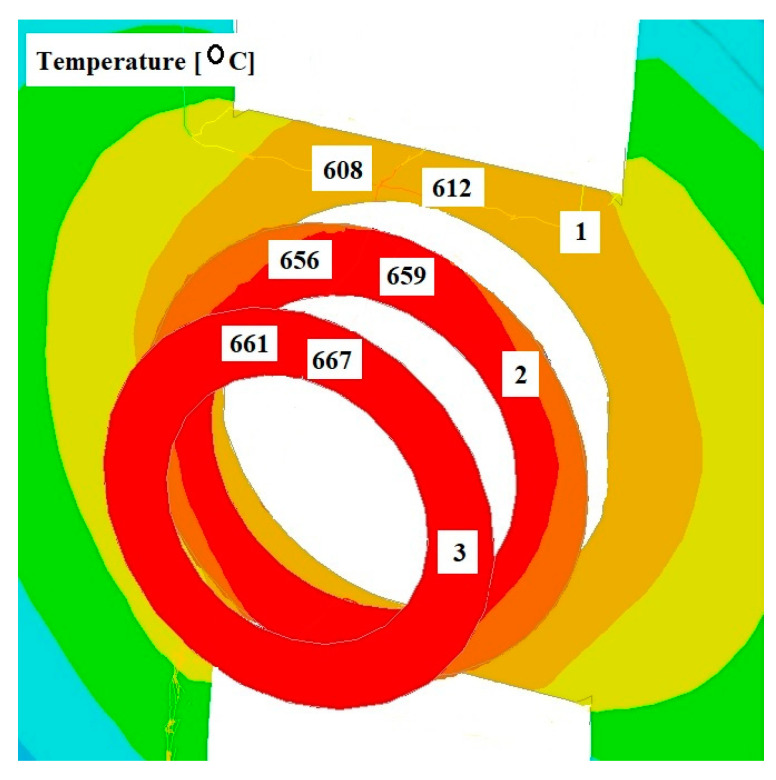
Distribution of temperature values on cross-sections of a charge extruded at 10 mm/s and at 600 °C.

**Figure 11:**
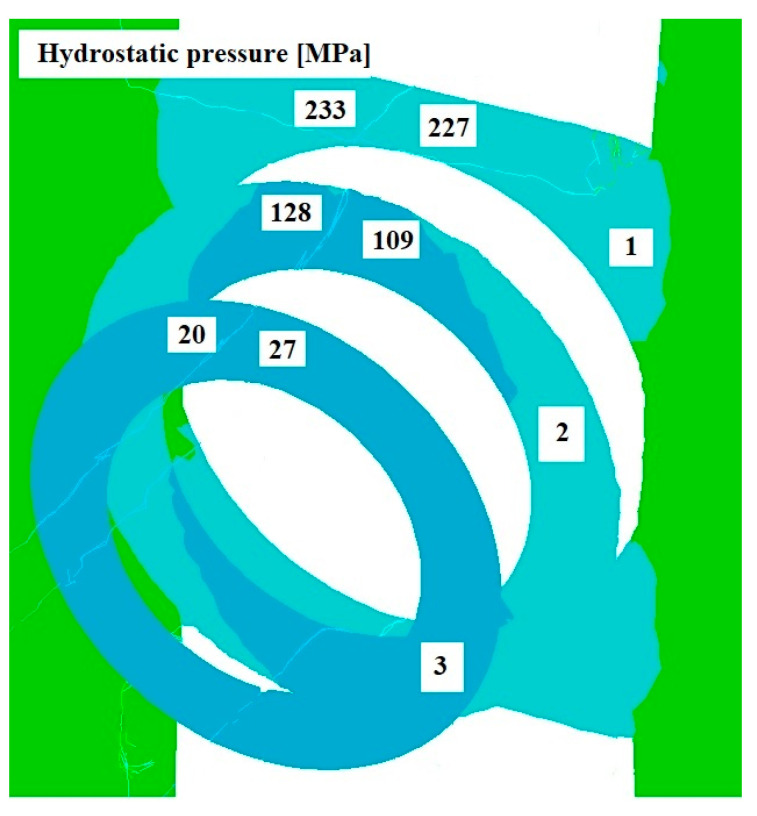
Distribution of hydrostatic pressure values on cross-sections of a charge extruded at 10 mm/s and at 800 °C.

**Figure 12:**
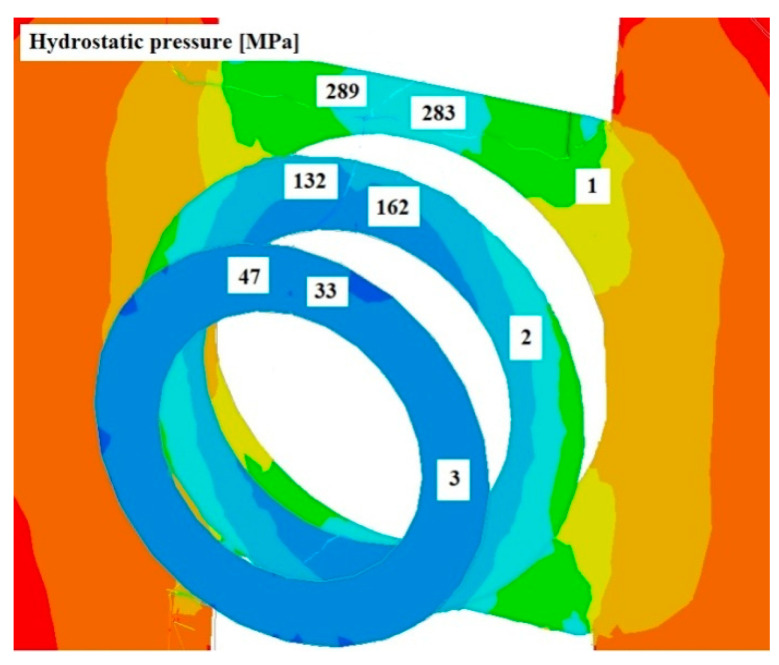
Distribution of hydrostatic pressure values on cross-sections of a charge extruded at 10 mm/s and at 700 °C.

**Figure 13:**
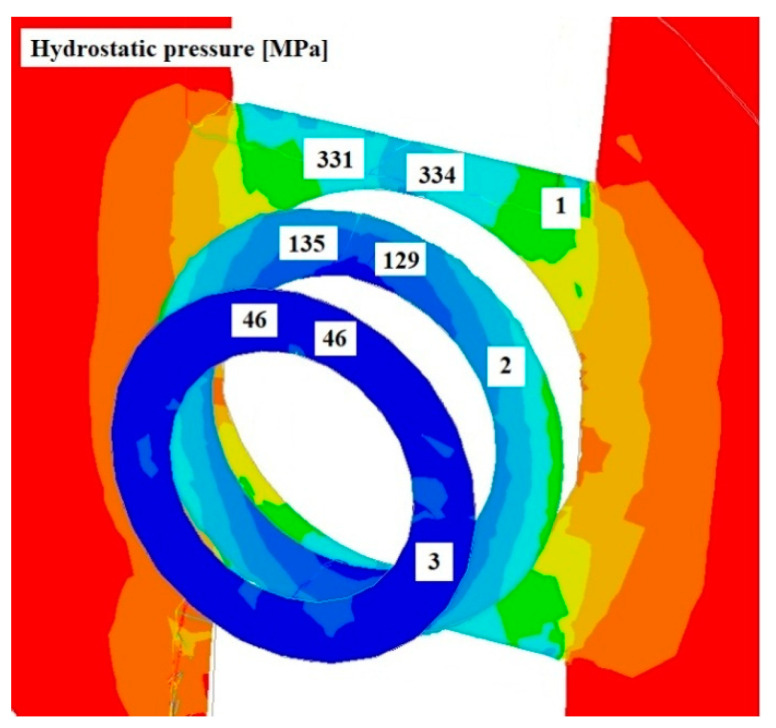
Distribution of hydrostatic pressure values on cross-sections of a charge extruded at 10 mm/s and at 600 °C.

Figure 8, Figure 9 and Figure 10 show the distributions of temperature values, while Figure 11, Figure 12 and Figure 13 show the distributions of hydrostatic pressure values obtained in simulations of the extrusion process of a pure anaerobic copper tube for three different initial charge temperatures: 600, 700, and 800 °C.

Three cross-sections, perpendicular to the material axis, were obtained at locations specific to the extrusion process: the centre of the welding chamber—1; the centre of the die deformation cavity—2; and the die output—3.

The data shown in Figure 8 and Figure 9 indicate that in the area of the welding chamber, a temperature drop was observed for initial charge temperatures of 800 °C and 700 °C, amounting to 25 °C and 60 °C, respectively. This decrease occurred due to the difference between the initial temperatures of the charge and the tools. For an initial temperature of 600 °C (Figure 10), a slight increase in the temperature of the extruded material, amounting to several degrees, was observed. The initial charge temperature of 600 °C was low enough to show the effect of the deformation process, which influenced this slight temperature increase. In the other two cross-sections (2 and 3), an increase in the temperature of the extruded material was observed for all cases analysed. In all cross-sections studied, the temperature distribution obtained is favourable for the bonding process of the material separated by the bridge during the extrusion of the copper tube.

Based on the analysis of the data shown in Figure 11, Figure 12 and Figure 13, it can be concluded that the highest hydrostatic pressure values were obtained for the charge with the lowest initial temperature. This is due to it overcoming the highest strain resistance due to the low initial temperature of the charge. For all cases analysed, the highest stress values were obtained in the area of the welding chamber, which is beneficial for the process, as these hydrostatic pressure values support the welding of the material. The physical tests carried out show that the welding of the material for the initial temperature values investigated occurred at hydrostatic pressure values in the range 50–64 MPa, as shown in Table 1. The hydrostatic pressure values obtained during the numerical modelling of the process exceeded those obtained in the physical tests by several times. 

## 5. Conclusions

Based on the analysis of the test results, the following final conclusions were drawn:

- The use of appropriate welding parameters makes it possible to obtain a qualitatively good bond of the material separated by the bridge of the die in the welding chamber of the bridge die;
- Both the hydrostatic pressure and the temperature of the charge have a significant influence on the copper welding in the extrusion process;
- Complete welding of the material during the extrusion process was achieved for a charge temperature higher than 600 °C and a hydrostatic pressure of 45–65 MPa;
- The results of the numerical simulations were confirmed by physical tests;
- Based on observations of the bonded material under the specified process parameters using a microscope, no discontinuities were observed and the microstructure of the material in the bonding zone consisted of newly formed fine grains.

Based on the analysis of the temperature and hydrostatic pressure results obtained from the numerical modelling, it can be concluded that for the initial charge temperature range analysed, bonding of the material separated in the bridge die does occur. The minimum charge temperature required to ensure full welding of the copper in the die welding chamber is 600 °C. The lowest charge temperature among those studied is simultaneously conducive to an increase in hydrostatic pressure in the material being deformed, which has a positive effect on the formation of a permanent bond in the weld zone.

Physical investigations of the thermo-mechanical parameters for achieving a full copper bond indicate that a good quality of bonding of the material separated by the bridging die will occur under the real conditions of the tube profile extrusion process. 

At this stage of the research, the authors have not carried out laboratory trials of the extrusion process of oxygen-free copper through a bridging die of the given shape. However, we plan to continue physical testing in this area.

## Figures and Tables

**Table 1:** Summary of the results of the physical modelling of the welding process of the copper specimens, together with the complementary results of the numerical simulations.

Temperature	Change in Specimen Length	Maximum Force at Upsetting (Physical Experiment)	Maximum Force at Upsetting (Numerical Simulation)	Hydrostatic Pressure in the Weld Zone	Condition of the Weld after Upsetting
°C	mm	kG	kG	MPa	-
520	10	1900	-	-	None
570		1850	1660	45–65	Partial
600		1800	1550	45–65	Partial
640		1500	1400	40–63	Full
660		1470	1320	40–62	Full
700		1100	-	-	Full
800		600	-	-	Full
600	5	1240	-	-	None
660		930	-	-	Partial

## Data Availability

The original contributions presented in the study are included in the article, further inquiries can be directed to the corresponding author.
